# The rise and fall of acute rheumatic fever and rheumatic heart disease: a mini review

**DOI:** 10.3389/fcvm.2023.1183606

**Published:** 2023-05-23

**Authors:** Yunmei Liang, Dingle Yu, Qinghua Lu, Yuejie Zheng, Yonghong Yang

**Affiliations:** ^1^ Department of Pediatrics, Beijing Chaoyang Hospital Affiliated to the Capital Medical University, Beijing, China; ^2^ Department of Respiratory Medicine, Shenzhen Children’s Hospital, Shenzhen, China; ^3^ Microbiology Laboratory, National Center for Children’s Health, Beijing Pediatric Research Institute, Beijing Children’s Hospital, Capital Medical University, Beijing, China

**Keywords:** rheumatic fever, rheumatic heart disease, group A *Streptococcus*, antibiotics, scarlet fever

## Abstract

**Introduction:**

The incidences of acute rheumatic fever (ARF) and rheumatic heart disease (RHD), which were leading causes of death in children in the 1920s, have decreased substantially. Considering the recent resurgence of scarlet fever and increased incidence of streptococcal pharyngitis in children, an investigation of the current status of ARF and RHD may be worthwhile.

**Objective:**

To summarize the prevalence trends, pathogenic factors, and prevention strategies for ARF and RHD in children.

**Methods:**

A selective search of literature published between January 1920 and February 2023 was done in PubMed, using the terms “acute rheumatic fever”, “rheumatic heart disease”, “group A *Streptococcus*”, “pharyngitis”, “pharyngeal tonsillitis”, “scarlet fever”, “impetigo”, “obstructive sleep apnea syndrome” and “child”.

**Results:**

Overcrowded homes and inadequate sanitation led to recurrent group A streptococcal infection, and the causal relationship between group A streptococcal infection and ARF/RHD was well established. Streptococcal infectious diseases, such as group A streptococcal pharyngeal tonsillitis, SF, impetigo, and obstructive sleep apnea syndrome, were associated with the occurrence of ARF and RHD. ARF and RHD were still prevalent in young people of developing countries and economically poor populations of high-income countries. Universal disease registration systems were critical to locating disease outbreaks, tracking disease transmission, and identifying high-risk populations. Four-level prevention strategies were effective in reducing the incidence and mortality of ARF and RHD.

**Conclusions:**

Registry and preventive measures for ARF and RHD should be strengthened in areas of dense population; poor sanitation; resurgence of SF; and high incidence of streptococcal pharyngitis, impetigo, and obstructive sleep apnea syndrome.

## 1. Introduction

Acute rheumatic fever (ARF) and rheumatic heart disease (RHD) are immune disorders caused by group A *Streptococcus* (GAS) infections; these conditions were the leading cause of death among children in the 1920s (1, 2). However, the improvement of economic and living conditions, as well as the widespread use of antibiotics, led to a marked decrease in the frequency of GAS infections, which was paralleled by a decrease in the incidence of ARF and RHD (3–6). By the 1980s, RHD among children had almost disappeared in developed countries of Europe and North America, but continued to cause a heavy disease burden in developing and low-income countries (7, 8).However, with the re-emergence of scarlet fever (SF) and the high incidence of streptococcal pharyngitis in children in recent years, is an increase in the incidence of ARF and RHD really far away?

This review was aimed at summarizing the changing epidemiology and potential risk factors and prevention strategies for ARF and RHD in children.

## 2. Objective and methods

In order to summarize the prevalence trends, pathogenic factors, as well as prevention and control measures of ARF and RHD in children, a selective literature search was performed for entries published between January 1920 and February 2023 in PubMed. The following search terms were used: “ARF”, “RHD”, “group A *Streptococcus*”, “pharyngitis”, “pharyngeal tonsillitis”, “scarlet fever”, “impetigo”, “obstructive sleep apnea syndrome”, and “child”.

## 3. Results and discussion

### 3.1. Epidemiology of ARF and RHD

ARF is an autoimmune reactive disease caused by untreated *Streptococcus* pyogenes, or GAS, infection in genetically susceptible hosts; it often occurs 2–3 weeks after GAS infection (2). The clinical manifestations of ARF include carditis, mono- or polyarthritis, Sydenham's chorea, erythema marginatum, and subcutaneous nodules, with secondary manifestations of fever, arthralgia, elevated levels of inflammatory markers [erythrocyte sedimentation rate (ESR), C-reactive protein (CRP)], and prolonged *P*-R interval (4, 9). There are 3 stages in the development of ARF:bacterial sore throat, ARF, and RHD (9). About 60% of individuals with ARF develop RHD within 10 years of the initial diagnosis, and recurrent ARF exacerbates existing heart damage (10).

RHD is one of the most common and preventable forms of acquired heart disease, and it is caused by chronic heart valve damage due to a single severe or multiple recurrent episodes of ARF (11). RHD can lead to complications such as heart failure, endocarditis, embolic stroke, and atrial fibrillation, which may be fatal in severe cases (9). RHD is the leading cause of morbidity and mortality in children aged 5–15 years (1).

ARF and RHD occur worldwide. Their incidence is influenced by various factors such as geographic location, climate, season, economic status, nutritional status, housing and sanitation, gender, GAS infection rate, ethnicity, and family susceptibility (2, 4). Factors such as cold weather, humidity; autumn, winter, and spring (especially spring) seasons; poverty; overcrowded households; and prevalence of GAS infection are associated with increased prevalence of ARF and RHD (2, 4). The prevalence of ARF is higher in girls than in boys (4, 10, 12). Pacific Islander children and indigenous Maori children have a high likelihood of developing ARF (5).

ARF and RHD were prevalent in the late 19th and early 20th centuries (4). In the 1920s, ARF was the leading cause of death among individuals aged 5–20 years old in the United States (7). Since the early 20th century, the incidence and prevalence of ARF and RHD have trended downward in developed countries, which is thought to be the result of improved living conditions and the use of antibiotics for the treatment of GAS infections. However, RF and RHD continue to remain as leading causes of morbidity and mortality in young people from developing countries. The World Health Organization cites overcrowding, poor housing conditions, inadequate nutrition, and lack of health care as reasons for the persistence of these disease in developing countries.

More than 15 million cases of RHD are estimated to occur worldwide, with 282,000 new cases and 233,000 deaths per year (13). During the 1920s and 1940s, the incidence of ARF and RHD in children was significantly reduced by measures such as changes in living conditions, nutritional support, and extended physical and psychological rest at the onset of the disease (14). With the use of penicillin in clinical practice after the 1940s, the incidence of ARF and RHD decreased further (5, 15). During the same period, primary and secondary prevention systems for ARF and RHD were established (6). In the 1970s, ARF and RHD were rare in most European countries and developed countries of North America (7). The incidence of RHD was almost zero in the 1980s (8). ARF can cause heart valve damage in severe cases, but remain asymptomatic (i.e., subclinical carditis) in some (16). Currently, subclinical carditis, or asymptomatic RHD, is considered as a manifestation of ARF, and early diagnosis and timely secondary prevention of asymptomatic RHD can prevent permanent valve damage due to recurrent ARF (17). The use of echocardiography and Doppler technology in clinical practice has improved the detection rate of RHD, allowing early diagnosis and intervention even in asymptomatic, latent RHD and thereby contributing to the reduction in morbidity and mortality associated with RHD (17). By the end of the 20th century, ARF and RHD had virtually disappeared from developed countries (18). However, they remained significant causes of morbidity and mortality in economically backward, developing, and war-torn countries and economically poor populations of high-income countries (19). For example, the incidence of ARF in New Zealand children has not declined significantly since the 1980s (20). Between 1985 and 2000, ARF and RHD had a resurgence in urban and middle-class white populations in Salt Lake City, Utah, and its mountainous region (15). During the past 20 years, Italy and the southern Central European country of Slovenia have shown signs of resurgence of ARF and RHD (21, 22). ARF and RHD remain a problem that cannot be ignored in economically developed countries such as Israel and Italy (23). The global prevalence of RHD increased by 70.49% between 1990 and 2019 (24). Africa and south Asia have large burden of ARF and RHD. According to the Global Burden of Disease Survey results, the age-standardized disability-adjusted life years rate for RHD was the highest among South Asian superregion, and Central Sub-Saharan Africa ranked the next highest in 2019 (25). Thus it is reasonable to infer that, the burden of ARF and RHD is evident in most parts of the world.

In China, the risk of ARF and RHD is medium to high (26). The incidence of ARF and RHD had peaked in the 1940s–1950s, but began to decline during the 1950s–1970s (26). Since then, the prevalence of ARF and RHD has varied across regions of China due to various reasons, including geography, climate, and economics. By the end of the 1980s, ARF and RHD had become rare diseases in Taiwan, China (27). However, by the early 21st century, there appeared to be a resurgence of ARF and RHD in children in Taiwan, China (28). Between 1969 and 1985, the hospitalization rate for ARF in Hong Kong, China, had decreased by 97%, and the hospitalization rate for RHD decreased from 6.3 to 3.0 per 1,000 hospitalized population (29).

Since the 1950s, the incidence of ARF and RHD in mainland China has been on a downward trend (28), decreasing from >100/100,000 in the 1970s–1990s to 5–20/100,000 in the 1990s–2010s (8), although there was an increase in 1960s–1970s (26). In the 1990s, the burden of ARF in mainland China was still heavy, and children comprised the majority of the population with ARF and RHD. In some areas, primary and secondary measures for the prevention of ARF and RHD had not been implemented, and the prevalence of ARF among primary and secondary school students aged 5–18 years was as high as 20.05/100,000 (30). In some regions, the level of control of ARF and RHD is only equivalent to that of Europe and the USA during 1950s–1960s (31). In the early 21st century, with the improvement of economic level, medical care, and housing conditions, the majority population of RHD patients had changed from children to adults and the elderly, but RHD remains the leading cause of valvular heart disease in mainland China (32). Since the nineties of the twentieth century, literature on RHD in Chinese children has become scarce, and many young doctors do not know about ARF and RHD.

### 3.2. Pathogenic factors of ARF and RHD

Acute pharyngitis is an acute, specific or non-specific inflammatory response of the pharyngeal mucosa, submucosa and lymphatic tissues and is a common disease that occurs in children between the ages of 5 and 15 years (1, 9). The main clinical manifestations are sore throat, painful swallowing and fever. GAS is the most common bacterial cause of acute pharyngitis in children, accounting for approximately 20%–30% of cases of acute pharyngitis in children (33). ARF in childhood is usually triggered by GAS infection of the pharyngeal tonsillitis, and RHD is a potential complication of untreated streptococcal pharyngeal tonsillitis (34). Approximately 616 million people worldwide suffer from acute streptococcal pharyngeal tonsillitis each year (13). ARF complications occur in about 5%–6% of the population (13). In Argentina, 18.1% of rural children are carriers of GAS infection in the throat (35). GAS is the pathogenic cause in 17.7% of the cases of acute pharyngitis among Tunisian children (36). Since 2014, the incidence of acute streptococcal pharyngeal tonsillitis and peri-tonsillar abscesses has spiked in England (37). In 2018, there was an outbreak of acute streptococcal pharyngitis among recruits in Quebec, Canada (38).

Impetigo is the most common bacterial skin infection in children aged 2–5 years, and GAS is one of the main causative agents (39). In New Zealand, studies have shown that GAS is the causative agent in approximately 12.7% of the cases of childhood impetigo (40). Global statistics from 2015 show that more than 162 million children in low- and middle-income countries have impetigo, with the median prevalence being the highest in children in warm and humid Oceania (29.7%); further, half of the children living in remote Aboriginal communities in northern Australia and Torres Strait Islanders develop impetigo (44.5%) (41). The median prevalence of impetigo in children from marginalized communities in high-income countries has been reported to be as high as 19.4% (42). During July–August 2018, there was an increase in the incidence of impetigo in Dutch children for unknown reasons (43).

Conventionally, ARF is not thought to occur after skin infections. However, contemporary studies have confirmed the association between impetigo and ARF and RHD. In Australia and New Zealand, where the prevalence of streptococcal impetigo is high and that of streptococcal pharyngitis is low, the prevalence of ARF and RHD is among the highest in the world (10, 39).

Obstructive sleep apnea syndrome (OSAS) is a common pediatric disorder characterized by recurrent events of partial or complete upper airway obstruction during sleep. The prevalence of OSAS is approximately 1%–5%, and it results in abnormal ventilation and sleep patterns (44). The peak age of prevalence is 5–8 years. Adenoids and tonsillar overgrowth are the most common causes of upper airway restriction during sleep. Tonsillectomy with or without adenoidectomy is the first-line treatment for OSAS. The correlation of ARF and RHD with enlarged tonsils has been frequently reported, and tonsillectomy is a treatment for ARF and RHD. In 2016, Viciani et al. found that GAS infection was strongly associated with the development of OSAS and that about 50% of children with OSAS carry GAS strains on their enlarged tonsils and had the M18 type of GAS strain that can cause ARF (45).

SF is a clinical syndrome associated with streptococcal pharyngeal tonsillitis that most often affects children aged 5–15 years, with clinical manifestations of fever, rash, streptococcal pharyngotonsillitis, and bayberry tongue (46). By the mid-20th century, SF had largely disappeared in developed countries (12). However, since the 2010s, SF has resurfaced in China, the United Kingdom, Germany, and Spain, among other countries (47). The relationship of epidemics of streptococcal infection to rheumatism has long been emphasized. SF is closely related to ARF and RHD; as early as 1902–1905, Galabin and Longstaff investigated the relationship between SF and ARF (2, 48). A recent history of SF was one of the conditions for GAS infection included in the Jones Criteria for the Diagnosis of ARF (1965 edition) (49).

### 3.3. Prevention and control strategies of ARF and RHD

ARF and RHD are potentially fatal diseases that are prevalent in socioeconomically deprived areas of the world, but they can be prevented and eradicated with a four-tier prevention and control strategies comprising primordial, primary, secondary and tertiary levels (50, 51).

Primordial prevention of ARF and RHD comprises measures aimed at improving the environmental, social, and economic conditions of people at risk and eliminating exposure to socioeconomic and environmental risk factors (50–52). ARF and RHD are often referred to as “diseases of poverty”. Environmental factors such as overcrowded homes and inadequate sanitation are fundamental reasons for the recurrence of GAS infections in childhood, while socioeconomic factors such as lack of medical resources, inadequate nutrition, unemployment, low income, overall socioeconomic status, low educational level, and social status are factors that contribute to the occurrence of ARF and RHD (52). Thus, primordial prevention refers to reducing the incidence of GAS infection, ARF, and RHD through interventions that target socioeconomic or environmental factors. Studies have shown that the incidence of GAS infection, ARF and RHD can be reduced by providing environmental health and educational support based on community needs through community health workers (51). The effectiveness of primordial prevention has also been confirmed by the results of ARF and RHD prevention and control in Europe and North America in the early 20th century (14). Primordial prevention should be part of a comprehensive strategy to eliminate RHD as a public health problem.

Primary prevention is the use of antibiotics to treat acute group A streptococcal infections in the absence of an effective vaccine to reduce the incidence of ARF (51–53). The clearance rates of penicillin, cephalosporins and macrolides for GAS infections have been reported to be 84.1%, 82.7%, and 71.7%, respectively. The eradication rate of erythromycin was about 80% for sensitive GAS strains and 60% for drug-resistant GAS strains (54). Primary prevention measures and control strategies of early detection and eradication of GAS infection have been shown to reduce the risk of ARF and RHD by 80% (53). Antibiotics are an important tool to combat GAS infections, and primary prevention is based on the early and correct diagnosis of acute GAS infections. However, viruses may be the actual causative agent of acute pharyngitis in approximately 10%–25% of school-aged children who test positive for GAS and have clinical symptoms (55). The reason for this false implication of GAS in viral infections is the presence of asymptomatic GAS colonization (or carriage) in the pharynx/tonsils of some school-aged children (56). GAS colonization (or carriage) is currently defined by a positive GAS pharyngeal swab culture that does not produce an ASO antibody response (57). The risk of colonization (or carriage) with GAS strains for transmission, the development of septic infection, complications of ARF, and the ability of antibiotic therapy to clear colonies of GAS strains still remain controversial (1, 35).This makes it difficult to implement primary prevention strategies for ARF and RHD.

Secondary prevention is a strategy to prevent the recurrence of ARF and the progression of RHD to the severe form (allowing the disease to subside) by continuing antibiotics in people who have had previous episodes of ARF or who already have RHD (58). Regular administration of benzylpenicillin G (BPG) reduces streptococcal pharynx/tonsil infections and ARF recurrence rates (9, 50). Early detection of ARF and use of antibiotics for secondary prevention is essential to combat RHD. However, a treatment strategy of intramuscular injection of BPG every 4 weeks for at least 5–10 years makes adherence a key determinant of success in the secondary prevention of ARF and RHD (59). Lack of funding, remoteness of facilities, lack of medical resources, fear of side effects, painful injections, and lack of awareness of the importance of treatment have been reported as the main reasons for poor adherence to secondary prevention (60). Inadequate adherence to secondary prevention measures increases the risk of recurrence of ARF and worsening of RHD after each recurrence of ARF (9, 60). Optimizing adherence and ensuring safe and adequate supplies of medications are key to the success of secondary prevention, and a delivery model with dedicated services, case management, and family support can improve adherence to secondary prevention in the ARF/RHD population (61).

Tertiary prevention strategies comprise the pharmacological and surgical management of RHD complications to reduce the morbidity and mortality associated with RHD (50, 51). Tertiary interventions for RHD include medical management of heart failure; surgical management of valvular lesions; and treatment of embolic stroke, infective endocarditis, and arrhythmic complications (50). Angiotensin-converting enzymes, diuretics, and fluid restriction are the mainstays of the treatment of heart failure (9, 62). Surgical treatment is the main treatment option required to rescue RHD patients with uncontrolled drug-associated congestive heart failure (63). Valve repair or valve replacement is the common surgical treatment for rheumatic valve disease, with valve repair being superior to valve replacement (64). Bipolar radiofrequency ablation is the most common surgical treatment for mitral valve disease and also an effective technique for the treatment of long-term persistent RHD-associated atrial fibrillation during mitral valve replacement (65). Percutaneous balloon mitral valvuloplasty is an effective treatment for isolated Mitral Stenosis(MS) or MS with mild degree of Mitral Valve Insufficiency (66). Atrial fibrillation is a common complication of RHD valve disease, and atrial fibrillation can cause heart failure, stroke, peripheral thromboembolism, and premature death. Early diagnosis and treatment of endocarditis and the use of anticoagulants to prevent and treat RHD-associated atrial fibrillation and prosthetic valve-related embolic disease are important components of the tertiary prevention strategy for RHD (64). RHD is the most serious sequela of ARF and is often a poor outcome of ineffective primordial, primary, and secondary prevention, and tertiary prevention has an important role to play in areas with a high prevalence of RHD (67).

Knowledge of the prevalence of pathogenic GAS strains can help in planning appropriate strategies for the prevention of ARF and RHD. Universal disease registration systems are crucial to locating disease outbreaks, tracking disease transmission, identifying high-risk populations, and making decisions about disease control. The global disease registry reporting system began in July 1837, and SF, ARF, and RHD are diseases that require registration and reporting (68). The prevalence of GAS infection-associated diseases has changed over time, and there are differences in the types of GAS infection-associated diseases registered in different countries and regions. For example, SF is a legally reported disease in many Asian countries and the UK, but not in the US (69). In view of the resurgence of SF and ARF in several countries or regions, some countries around the world have started developing measures to strengthen the surveillance of GAS infection and thereby prevent progression to RHD—a fatal complication associated with GAS infection (23, 69). Regions with high incidence of RHD, such as Australia, have accumulated a rich experience in the management of ARF and RHD registration and reporting systems (10).

## 4. Conclusions

GAS is a pathogen that has re-emerged worldwide. It can cause a variety of superficial diseases such as pharyngitis, SF, impetigo, and OSAS, which are recognized causative factors of ARF and RHD. Prevention of ARF and RHD and registry of incident cases should be strengthened in areas of dense population and poor sanitation, in areas where the incidence of streptococcal pharyngitis and impetigo remains high, and in areas where SF and invasive streptococcal infections are resurgent.
